# Associations Between Gut Microbiota and Mitochondrial Metabolites, with Growth Differentiation Factor-15 as a Marker of Oxidative Stress in Heart Failure vs. Healthy Ageing

**DOI:** 10.3390/antiox15020199

**Published:** 2026-02-02

**Authors:** Konstantinos Prokopidis, Adam Burke, Beyza Gulsah Altinpinar, Sima Jalali Farahani, Omid Khaiyat, Gregory Y. H. Lip, Rajiv Sankaranarayanan, Vanja Pekovic-Vaughan, Howbeer Muhamadali, Masoud Isanejad

**Affiliations:** 1 Department of Musculoskeletal Ageing and Science, Institute of Life Course and Medical Sciences, University of Liverpool, Liverpool L7 8TX, UK; 2 Liverpool Centre for Cardiovascular Science at University of Liverpool, Liverpool John Moores University and Liverpool Heart & Chest Hospital, Liverpool L7 8TX, UK; 3 Centre for Metabolomics Research, Department of Biochemistry, Cell and Systems Biology, Institute of Systems, Molecular and Integrative Biology, University of Liverpool, Liverpool L69 7BE, UK; 4 Department of Cardiovascular and Metabolic Medicine, Institute of Life Course and Medical Science, University of Liverpool, Liverpool L7 8TX, UK; 5 School of Health and Sport Sciences, Liverpool Hope University, Liverpool L16 9JD, UK; 6 Liverpool University Hospital Foundation Trust Liverpool, Liverpool L7 8YE, UK

**Keywords:** heart failure, GDF-15, gut microbiota, metabolites, sarcopenia

## Abstract

Growth differentiation factor-15 (GDF-15) is an established marker of oxidative stress and a general stress-response mitokines. In this study, we aim to investigate the association of GDF-15 with the metabolic signature of gut and mitochondrial activity in HF and ageing population. A total of 25 HF (67.9 ± 10.0 years) and 29 age-matched healthy participants (HPs) (67.8 ± 11.1 years) were recruited and underwent detailed body composition assessment via dual X-ray absorptiometry; total fat mass and appendicular lean soft tissue index (ALSTI/body mass index (BMI)) were calculated. Utilizing semi-targeted Gas Chromatography–Mass Spectrometry on fasting plasma, a panel of gut microbial-derived (e.g., hippuric acid, indole derivatives, and sarcosine) and tricarboxylic acid cycle metabolites was identified. Results showed higher GDF-15 tertiles were associated with greater HF prevalence, fat mass, NT-proBNP, and TNF-α (*p* < 0.05). Gut-derived metabolites exhibited phenotype-specific patterns; 3-hydroxyindole predicted higher fat mass in HP; hippuric acid was inversely related in HF; and sarcosine correlated with GDF-15 only in HP. In HF, GDF-15 was strongly driven by pyruvic and fumaric acid, indicating disease-specific mitochondrial stress. In conclusion, these observed associations could be evaluated in future mechanistic studies as sensitive biomarkers of systemic oxidative stress markers, informing potential microbiome-targeted therapeutic avenues.

## 1. Introduction

Heart failure (HF) is a systemic condition that extends beyond impaired cardiac pump function [1]. The pathophysiology of HF is multifactorial, and the contribution of oxidative stress and reactive oxygen species (ROS) remains to be understood. Although preclinical models can provide insights, they may not fully recapitulate the biological complexity and heterogeneous pathogenesis observed in humans. Measuring biomarkers of oxidative stress, including members of the transforming growth factor-β (TGF-β) family, together with metabolic signatures may offer new insights into the interconnected pathways driving HF and reveal oxidative stress-induced mechanistic links that have previously been overlooked. Particularly, elevated growth differentiation factor-15 (GDF-15) and related metabolites may reflect activation of stress-responsive pathways triggered by mitochondrial dysfunction and oxidative damage [2,3]. Their induction alongside oxidative stress markers may govern tissue resilience and systemic metabolic homeostasis. Among these, the gut has emerged as an important interface [4,5,6] where reduced perfusion, congestion, and altered motility in HF can disrupt the intestinal environment and reshape the gut microbiota, influencing host metabolism [7]. This two-way interaction between the gut and the circulating metabolome may contribute to the progression of HF through oxidative stress, raising the possibility that gut microbial-altered microenvironments could contribute to cardiac dysfunction [8], given that microbial metabolites may enter the bloodstream and act on distant tissues including the heart [5,9,10,11]. Additionally, gut-derived metabolites could reflect dietary patterns, microbial activity, and intestinal health, providing a sensitive non-invasive window into gut function that may help clarify mechanisms relevant to cardiometabolic disease [12,13].

Under normal physiological conditions, mitochondria act as the primary source of ROS, generated as by-products of aerobic respiration and contributing to endogenous oxidative stress. While capturing meaningful and accurate measurements of mitochondrial redox stress remains a substantial challenge, mitochondrial metabolites are shown as a strong indicator of its homeostasis [14]. Considering that obesity is closely linked to alterations in oxidative stress [15], energy homeostasis, and the gut microbiota [13,16], it is important to understand whether gut-derived metabolites relate to fat mass and muscle quality, as indicated by the appendicular lean soft tissue index to body mass index ratio (ALSTI/BMI) in HF [17].

GDF-15 may serve as a key biomarker and mediator in HF. Produced primarily by cardiomyocytes under cardiac stress, such as ischemia, pressure overload, inflammation, or oxidative damage, GDF-15 levels may markedly elevate in HF, reflecting myocardial injury severity. In HF, elevated circulating GDF-15 correlates strongly with adverse outcomes, including progression to severe systolic dysfunction, hospitalizations, and mortality. In addition, GDF-15 may be implicated in muscle-wasting disorder, including cachexia, and involuntary rapid weight loss accompanied by decreases in both muscle mass and adipose tissue. Previous cohorts have found an inverse association between circulating GDF-15 and protein intake in HF [18], as well as sarcopenia and frailty in geriatric patients [19], reiterating its prominent link with adverse cardiac and muscle health-related outcomes.

In this study, we initially aimed to investigate the associations of GDF-15 with tumour necrosis factor-alpha (TNF-α), cardiac stress as reflected by NT-proBNP, and body composition in an ageing and heart failure (HF) population. Secondly, given the mitochondrial and gut-related metabolic abnormalities commonly observed in HF, we conducted a semi-targeted analysis of a predefined panel of gut- and mitochondria-derived metabolites in patients with chronic HF and in aged healthy participants (HPs). Our objective was to examine how these metabolites relate to key clinical and biochemical markers, including fat mass, ALSTI/BMI, inflammation (TNF-α), cardiac stress (NT-proBNP), and stress-response signalling as reflected by GDF-15.

## 2. Materials and Methods

### 2.1. Study Population

Community-living patients with chronic HF were recruited by reviewing medical records from Aintree Hospital at the Liverpool Hospital Foundation Trust from November 2023 until August 2024. HF patients were diagnosed by cardiologists according to the European Society of Cardiology HF guidelines [20]. The NT-proBNP threshold was adapted from the National Institute for Health and Care Excellence, and echo ejection fraction was estimated using the European Society of Cardiology HF guidelines. HPs were recruited from the general healthy population, with (self-reported) absence of any chronic disease. The study was registered at Clinicaltrials.gov NCT06217640 (released date 10 March 2023). Detailed methods of the study have been made available online [21].

Participants were included if they (i) had a clinical diagnosis of HF using left ventricular ejection fraction (LVEF%) for classification, (ii) had a BMI between 18 and 30 kg/m^2^, (iii) were aged >50 years old, (iv) were on optimal medical therapy for minimum 3 months before participating in the study, and (v) were able to provide informed consent and walk a minimum of 10 m (with or without a walking aid). HPs were eligible if they were under control for hypertension or hypercholesterolemia without history of chronic conditions. Participants were excluded if they (i) had a recent treatment (within 3 months) of antibiotics or probiotics, (ii) had comorbidities including chronic irritable bowel syndrome, kidney failure, late-stage cancer, or concurrent infections, (iii) were being treated with immunosuppressive medications, and (iv) received cardiac resynchronization therapy within the past 6 months.

### 2.2. Gas Chromatography–Mass Spectrometry (GC–MS) Analysis

Blood samples were collected in the morning after an overnight fast. Samples were centrifuged at 2000 *g* for 15–20 min at 4 °C, aliquoted into two 2 mL microcentrifuge tubes, and stored at −80 °C. GC–MS was completed at the Centre for Metabolomics Research, University of Liverpool. Plasma samples were deproteinized in a single batch by adding 300 μL ice-cold acetonitrile/methanol (1:1) to 100 μL homogenized plasma spiked with deuterated internal standards, vortexed for 15 s, and centrifuged at 30,130 *g* for 15 min. The supernatant was transferred into microcentrifuge tubes (Eppendorf, Stevenage, UK) and lyophilised for 16 h in a vacuum centrifuge (Savant SpeedVac SPD130DLX, Savant vapour trap RVT5105) (Fisher Scientific, Loughborough, UK). Pooled quality control (QC) and process blanks were prepared in tandem. Procedures for GC–MS analysis were adapted from the method presented by Dunn et al. [22]. Lyophilised samples, QCs, and blanks were derivatized in a two-step process, randomized, and analyzed via autosampler injection with GC–MS. An Agilent 7250 GC-ToF-MS equipped with an HP-5 ms (length 30 m, inner diameter 0.25 mm, and film thickness 0.25 µm) capillary column (Agilent, Cheadle, UK) was utilized, with an oven temperature gradient of 70 °C, 4 min hold, 20 °C/min to 300 °C, 4 min hold, and 300 °C transfer line temperature. MassHunter Acquisition software v.12.2 was set to automatically recalibrate the mass spectrometer following every four sample injections. Data were processed through spectral deconvolution, alignment, blank subtraction, and retention index assignment. Peak annotation was performed using an in-house spectral library concatenated with purchased external libraries for untargeted discovery. Signal correction and quality assessment across the batch runs were carried out using pooled QC samples. To identify gut-derived and mitochondrial metabolites, we performed a literature search to identify gut-derived metabolites that are present in plasma and used an online atlas (https://gutsyatlas.serve.scilifelab.se/app/gutsyatlas (accessed on 1 October 2025)) for further validation. For metabolites of tricarboxylic acid (TCA) cycle, we matched our data with those published [14].

### 2.3. Enzyme-Linked Immunosorbent Assay

We have utilised Thermofisher enzyme-linked immunosorbent assay (ELISA) to quantify GDF-15 [Human-GDF15-ELISA-Kit/EHGDF15X5] and TNF-α [Human-TNF-alpha-ELISA-Kit/BMS223-4] [Thermo Fisher Scientific, Cat. No. KAC1751] at the GCR lab at the Universiyt of Liverpool, Liverpool, UK. Standards, controls, and samples were plated in duplicates on 96-well microplates precoated with capture antibodies. After incubation with biotin conjugate and horseradish peroxidase-conjugated streptavidin, tetramethylbenzidine substrate was added. Absorbance was measured at 450 nm using a microplate reader. Biomarker concentrations were determined by interpolating absorbance values against standard curves generated with known concentrations.

### 2.4. Assessment of Body Composition

Dual X-ray absorptiometry (DXA) (GE Lunar Prodigy; GE HealthCare, Chicago, IL, USA) was used to assess body composition. Dual-energy X-ray absorptiometry (DXA) is vital for body composition assessment because it provides precise, reproducible measurements of fat mass, lean mass, and bone mineral content. DXA is non-invasive, fast, and standardized, enabling reliable monitoring of health, ageing, disease progression, and responses to nutrition or exercise interventions across diverse populations in research, clinical practice, and sport. Appendicular lean soft tissue (ALST) was calculated by summing lean mass from arms and legs, excluding fat and bone, which was used to estimate ALST index (ALSTI). ALSTI was calculated based on ALST divided by height (kg/m^2^). Measuring appendicular lean soft tissue is important because it reflects skeletal muscle mass and predicts strength, mobility, metabolic health, sarcopenia risk, and functional outcomes in ageing [23].

Lifestyle factors

Self-reported physical activity levels were assessed using the International Physical Activity Questionnaire (IPAQ), including moderate and vigorous physical activity. Additionally, activity was expressed in metabolic equivalent (MET) minutes per week by multiplying the number of daily activity minutes by the number of weekdays (score of 3.3 for walking, 4.0 for moderate activity, and 8.0 for vigorous activity). The Sarcopenia Quality of Life (SarQoL) questionnaire containing 22 questions regarding physical health, mental health, locomotion, body composition, functionality, activities of daily living, leisure activities, and fears was utilized to assess quality of life. Recorded responses were self-reported, and scores were transformed to a total score of 0 (lowest quality of life) to 100 (highest quality of life).

### 2.5. Statical Analyses

Baseline characteristics were compared across tertiles of GDF-15 using ANOVA for continuous variables and chi-square tests for categorical variables, to assess differences between groups at baseline. Further, regression analyses and Spearman correlation were conducted to estimate associations between metabolites, GDF-15, and body composition measures, and to inform a hypothesis-testing step in high-dimensional data, as previously established approach [24]. A *p* value of <0.05 was used to determine statistical significance, and all analyses were conducted using SPSS 22.0.

## 3. Results

Baseline characteristics of the study population stratified by GDF tertile groups are presented in Table 1 and Table S1. Age increased across GDF groups (mean age 64.4 ± 10.4, 67.5 ± 10.7, and 70.9 ± 9.9 years in tertile 1, 2, and 3, respectively), although the difference did not reach statistical significance (*p* = 0.244). The distribution of HP/HF phenotype was reduced significantly towards the higher tertiles (*p* = 0.029), with a higher proportion of HF classifications observed in tertile 3. The number of comorbidities was significantly higher in tertile 3 compared with tertiles 1 and 2 (*p* = 0.006). BMI differed across groups (*p* = 0.039), with the highest BMI observed in tertile 3. Total fat mass increased significantly with GDF-15 levels (*p* = 0.030), and total lean mass and total fat-free mass did not differ significantly between groups (*p* = 0.520 and *p* = 0.535, respectively). In addition, the higher level of GDF-15 was associated with worsened Sarcopenia Quality of Life score (*p* = 0.001)

**Figure 1:**
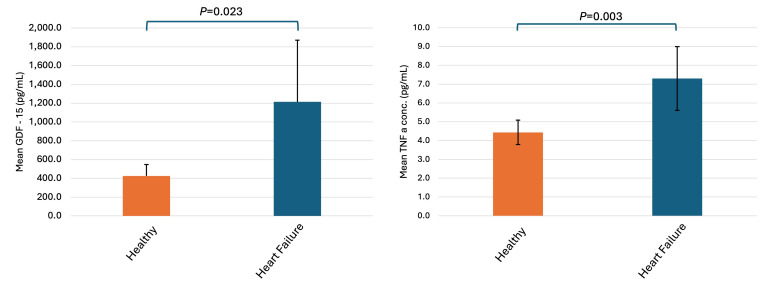
Concentrations of GDF-15 and TNF-α compared between healthy and 25 heart failure and 29 healthy participants; comparison between group was performed using independent sample *t*-test.

**Figure 2:**
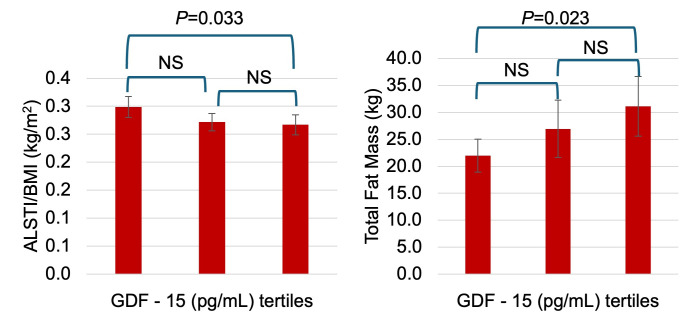
The distribution of fat mass and appendicular lean soft tissue index is stratified by GDF-15 tertiles. Analyses were performed using ANOVA with post hoc comparisons conducted using Fisher’s Least Significant Difference (LSD) tests. NS, not significant *p* > 0.050.

**Figure 3:**
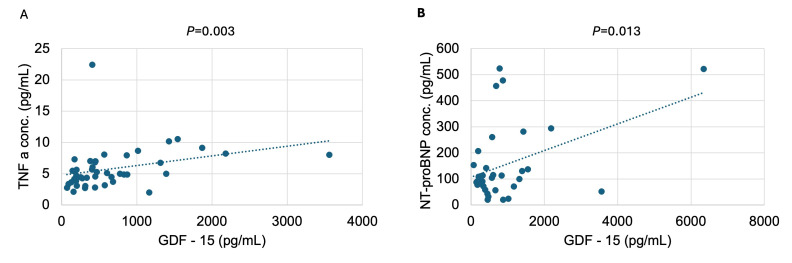
Regression plot between GDF-15 with (**A**) TNF-*α* and (**B**) NT-proBNP levels in total population.

ALSTI and ALSTI/BMI were largely comparable between groups, although ALSTI/BMI demonstrated a statistically significant between-group difference (*p* = 0.028). NT-proBNP concentrations were substantially higher in tertile 3 compared with groups 1 and 2 (*p* = 0.029). Similarly, TNF-α concentrations differed significantly across GDF groups (*p* = 0.029), with the highest levels observed in tertile 3. Overall, participants in higher GDF groups exhibited a phenotype characterized by greater disease burden, higher adiposity, and elevated inflammatory and cardiac stress biomarkers, while muscle mass indices remained largely similar across groups. Figure 1 shows the elevated levels of GDF-15 and TNF-α in HF compared to HP; Figure 2 shows the distribution of total fat mass and ALST/BMI across the GDF-15 tertiles; and Figure 3 indicates the association of GDF-15 with higher levels of NT-proBNP in the total population.

### 3.1. Association of Gut-Derived Metabolites with GDF-15

In HP, sarcosine was positively associated with GDF-15 (β = 0.466, *p* = 0.025), which was not observed in the HF group. Sarcosineamide, indole-3-hydroxy, indole-3-acetic acid, and hippuric acid demonstrated no significant associations with GDF-15 in either phenotype group (Table 2).

### 3.2. Association of Gut-Derived Metabolites with Body Composition

In the HP cohort, 3-hydroxyindole was a significant positive predictor of total fat mass (β = 0.396, *p* = 0.041) (Table 3 and Table 4). No significant associations were observed for indole-3-acetic acid, sarcosineamide, or hippuric acid in HP. In the HF cohort, hippuric acid emerged as a significant negative predictor of fat mass (β = –0.410, *p* = 0.047). 3-hydroxyindole only demonstrated a non-significant trend toward a positive association in HF (β = 0.364, *p* = 0.081). Sarcosine and sarcosineamide did not significantly predict fat mass in either group.

### 3.3. Association of Mitochondrial Metabolites with GDF-15

**Figure 4:**
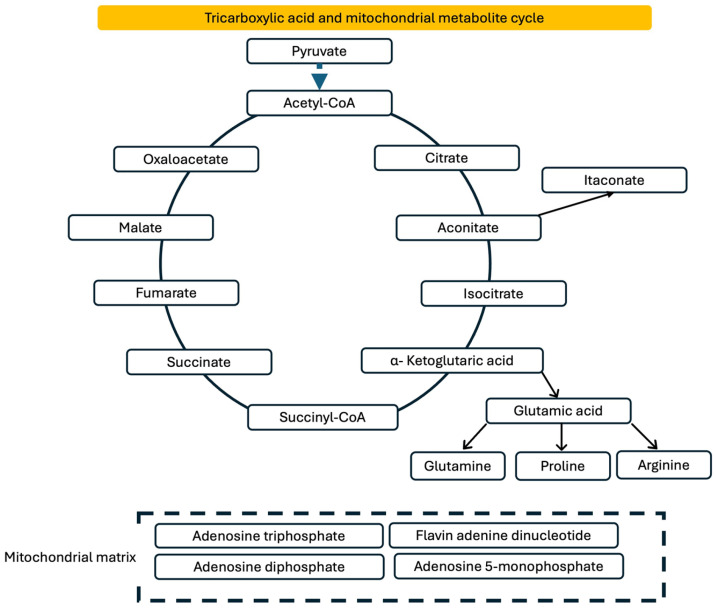
Presentation of TCA cycle metabolites.

Across the range of the TCA cycle (Figure 4) and related mitochondrial metabolites examined, only pyruvic acid and fumaric acid showed meaningful associations with circulating GDF-15, and these patterns differed between HP and HF groups (Table 5). In the HP group, pyruvic acid was a significant predictor of GDF-15 (β = 0.47, *p* = 0.024), whereas fumaric acid, citric acid, glutamic acid, 2-oxoglutaric acid, malic acid, and proline were not associated with GDF-15. In the HF group, both pyruvic acid (β = 0.70, *p* < 0.001) and fumaric acid (β = 0.73, *p* < 0.001) were strong predictors, together explaining a notable proportion of the variability in circulating GDF-15. None of the remaining metabolites showed statistically significant associations with GDF-15 in either group. Overall, the results suggest that mitochondrial carbon flux intermediates upstream in the TCA cycle, particularly pyruvate and fumarate, are more tightly coupled to GDF-15 concentrations in individuals with HF vs. HPs.

Across both HP and HF groups, none of the mitochondrial metabolites tested—pyruvic acid, fumaric acid, citric acid, glutamic acid, or malonic acid—were significantly associated with ALSTI/BMI. In the HP group, several metabolites showed small-to-moderate standardized effects (e.g., citric acid: β = 0.26; malonic acid: β = 0.24), but none reached statistical significance. The associations were even weaker in the HF group, with all β coefficients below 0.22 and corresponding *p*-values above 0.05. Together, these findings indicate that mitochondrial carbon intermediates do not independently explain variability in ALSTI/BMI in either HP or those with HF (Table 6).

Across both HP and HF groups, none of the mitochondrial metabolites analyzed—pyruvic acid, fumaric acid, citric acid, glutamic acid, 2-oxoglutaric acid, or malonic acid—were significantly associated with total fat mass. Although several metabolites showed small effect sizes, none reached statistical significance. In the HP group, pyruvic acid showed the largest effect (β = 0.33, *p* = 0.098), but this was still below the threshold for significance. In the HF group, similar small effects were observed, with all β values below 0.23 and all *p*-values above 0.29. Overall, these findings indicate that circulating mitochondrial metabolites did not meaningfully contribute to variations in total fat mass in either HP or those with HF (Table 7).

## 4. Discussion

Although the role of GDF-15 in metabolic stress and ageing is increasingly recognized, direct clinical studies linking GDF-15 with inflammatory and cardiac stress hormones (NT-proBNP), as well as metabolic status captured through mitochondrial and gut-derived metabolites in HF, are lacking. Chronically elevated GDF-15 in humans is associated with oxidative stress and catabolic state (e.g., cachexia) [25]. Our study aligns with the prior literature suggesting that GDF-15 is elevated in chronic HF [2,26,27], though our work demonstrates that this association is stronger with increased fat mass, implying a link to systemic proinflammatory and oxidative stress responses in adipose tissue [3,19,27]. The observed association of higher circulating GDF-15 with TNF-α and NT-proBNP provides important new insight into the integrated biology of inflammation, tissue stress, and cardiometabolic dysfunction. These findings align with my prior meta-analysis demonstrating that muscle weakness in HF is associated with elevated system cardiac and catabolic stress [28]. Clinically, this is highly important because it may position GDF-15 as a potential biomarker that captures overlapping risk domains (low-grade inflammation, myocardial stress, and adverse metabolic remodelling), which are central to oxidative stress of ageing and cardiometabolic disease progression. Overall, those with elevated GDF-15 had higher fat mass and lower indication of lean mass, while HF had lower ALSTI/BMI and higher fat mass compared to HP.

Differential associations between panels of gut-derived metabolites and key pathophysiological markers emerged when HPs were compared with patients with HF, highlighting phenotype-specific metabolic reprogramming. Certain gut bacteria can metabolize glycine and choline to sarcosine, which may secondarily influence sarcosine availability [29,30]; they were positively associated with circulating GDF-15 exclusively in the HP cohort, a relationship that was absent in HF patients. Hippuric acid was negatively associated with fat mass, accompanied by a borderline positive trend for 3-hydroxyindole in HF. Hippuric acid has been previously linked to reduction in visceral adipose tissue and BMI in a randomized controlled trial [31,32]. HF had elevated fat mass compared to HP, and hippuric acid was associated with less fat mass, suggesting that decline in metabolites can be an obesity-related biomarker. Currently, data regarding the role of 3-hydroxyindole in human are limited and our observed association may be explained by different microbiome activity in the HP group. Other gut metabolites, including indole-3-acetic acid and indole-3 propionic acids, showed no significant associations with either GDF-15 or fat mass in either group. Indole-3 propionic acid has been associated with HF particularly in preclinical studies [10,33]; in the current study, indole-3 propionic acid was lower in HF with non-significant trend (*p* from *t*-test = 0.076). The present metabolite associations could therefore inform on the hypothesis that metabolic signalling such as microbial metabolites may act as a network of biological markers when GDF-15 is physiologically elevated.

Mitochondrial stress and dysfunction are considered hallmarks of HF [34,35]. However, the multifaceted regulation of mitochondrial structure and function is poorly understood, especially in the context of age-dependent HF [36]. In the current study, GDF-15 showed positive associations with pyruvic and fumaric acid in HF, suggesting that mitochondrial substrate flux and redox imbalance may strongly influence stress signalling. These associations were absent in HP, which would be expected as these biological dysregulations are more pronounced in HF. Notably, altered TCA cycle metabolites are significantly associated with a protein hyperacetylation pattern [36] and mitokines signalling, which may participate in a bidirectional relationship [37,38]. A hypothesis may be that in HF mitokines such as GDF-15 are sent to other tissues, leading to cachexia and other skeletal muscle diseases, which are common in HF [39]. While the association of GDF-15 with muscle values was determined in our study and others [19], the association for mitochondrial metabolites with ALSTI/BMI or total fat mass in either group were not significant. While these may be due to sample size, it may be that fluctuations in circulating TCA cycle intermediates are more measurable when combined with a bigger contributor to chronic determinants such as physical inactivity levels or endocrine regulation in disease states [35]. Although previous research has suggested changes in TCA cycle, particularly in the context of cardiac energy metabolism, our results support a model in which GDF-15 may serve as a sensitive downstream marker of metabolic stress in HF.

Clinical relevance

Considering that elevated circulating GDF-15 strongly associated with key markers of cardiac stress (e.g., NT-proBNP) and inflammation (e.g., TNF-α), reflecting an amplified systemic response to myocardial injury and oxidative burden, its integration in clinical practice could provide further insights on potential adverse outcomes, including mortality, hospitalizations, and cardiac remodelling, adding to the prognostic value of NT-proBNP. Mechanistically, the positive GDF-15 association with TCA cycle intermediates (pyruvic and fumaric acid) in HF may indicate mitochondrial stress via redox imbalance and substrate flux dysregulation, supporting GDF-15 as a potential downstream mediator of the bidirectional signalling that may promote systemic catabolism, including skeletal muscle wasting and cachexia, conditions that may exacerbate HF progression via lower ALSTI/BMI. Overall, these observations highlight the potential of GDF-15 to aid in stratifying risk by encompassing inflammation, myocardial strain, and mitochondrial damage central to HF progression, which may be explained, in part, by microbial metabolites. However, larger trials to validate its utility for personalized monitoring and therapy in advanced HF are warranted.

Strengths and limitations

Our study has limitations, including the small sample size and the cross-sectional design, which limits causal inference and precludes bidirectional exploration, which is also a constraint on *p*-values to not be adjusted for false discovery rate. While being cross-sectional, and that associations are based on correlation coefficients, our results provide novel hypothesis for future larger studies and mechanistic intervention. Also, metabolic profiles are influenced by a wide range of lifestyle factors, including diet and medications. We did not incorporate microbiome data, which could have provided further insight, particularly into the gut microbiome and gut-derived metabolite content. Despite these limitations, and relative to the current state of the literature, our study provides important data on body composition and clinical biomarkers that have been lacking in previous studies. Furthermore, comorbidities and concomitant medications may confound metabolomic profiles, particularly in the relationship between gut-related metabolites and GDF-15 in heart failure. Validation of the metabolic pathways identified in this study will require future extensive in vivo and in vitro investigations, as well as larger clinical trials. Nevertheless, association-based findings such as ours represent an important first step in generating hypotheses and guiding future mechanistic research.

## 5. Conclusions

In HF, GDF-15 is strongly associated with pyruvate and fumarate, indicating mitochondrial substrate flux and redox imbalance stress signalling, which suggest elevated global and mitochondrial stress. Metabolomics in cardiovascular and redox research reveals metabolic pathways, oxidative stress signatures, and biomarkers, improving disease prediction, mechanistic understanding, and personalized prevention and treatment strategies. Larger and longitudinal studies are presently warranted to validate these results.

## Figures and Tables

**Table 1:** Bassline characteristics stratified for GDF-15 tertile.

Variable	Tertile 1 (71.2–313.4)	Tertile 2 (334.2–661.2)	Tertile 3 (683.4–3556.9)	*p*-Value
HP/HF	11/4	9/6	4/11	0.029
Age (Years)	64.40 (10.43)	67.47 (10.72)	70.87 (9.95)	0.244
Number of Comorbidities	0.40 (1.06)	1.08 (1.71)	2.60 (2.41)	0.006
Systolic BP (mmHg)	131.67 (11.19)	127.80 (20.40)	124.73 (12.93)	0.471
Diastolic BP (mmHg)	78.47 (8.54)	75.73 (10.56)	72.07 (13.23)	0.286
Sarcopenia Quality of Life score	79.7 (12.1)	76.4 (9.2)	59.7 (20.5)	0.001
Physical Activity Score	2.75 (0.5)	2.0 (0.6)	1.5 (0.5)	0.019
Body Mass Index (kg/m^2^)	25.05 (2.77)	27.67 (5.26)	29.79 (6.10)	0.039
Total Fat Mass (kg)	22.01 (5.93)	26.94 (9.93)	31.15 (10.72)	0.030
Total Lean Mass (kg)	46.35 (10.32)	48.15 (10.99)	50.57 (8.82)	0.520
ALSTI (kg/m^2^)	7.50 (1.33)	7.43 (1.38)	7.82 (1.13)	0.682
ALSTI/BMI	0.299 (0.036)	0.272 (0.027)	0.267 (0.035)	0.028
Total Fat-Free Mass (kg)	48.97 (10.86)	50.89 (11.62)	53.32 (9.19)	0.535
NT-proBNP (pg/mL)	109.14 (41.14)	90.55 (71.07)	228.95 (192.76)	0.029
TNF-α (pg/mL)	4.04 (1.35)	6.65 (4.61)	6.92 (2.53)	0.029

ALSTI: appendicular lean soft tissue index to body mass index; data are presented as mean (SD) or *n* (%), as appropriate. Group differences were assessed using one-way ANOVA for continuous variables and χ^2^ (chi-square) tests for categorical variables.

**Table 2:** Unadjusted linear regression models for association between gut-related metabolites and GDF-15 (pg/mL).

Predictor	Group	β (Standardized)	*p*-Value
Sarcosinamide	HP	–0.003	0.988
	HF	–0.036	0.878
Sarcosine	HP	0.466	0.025
	HF	0.191	0.407
Indole-3-acetic acid	HP	0.083	0.748
	HF	0.493	0.023
Indole-3 propionic acid	HP	0.023	0.362
	HF	0.064	0.234
3-hydroxyindole	HP	0.232	0.287
	HF	–0.111	0.631
Hippuric acid	HP	–0.024	0.913
	HF	0.017	0.943

**Table 3:** Unadjusted linear regression models for association between gut-related metabolites with total fat mass (kg).

Predictor	Group	β (Standardized)	*p*-Value
Sarcosinamide	HP	–0.023	0.911
	HF	0.068	0.752
Sarcosine	HP	0.344	0.079
	HF	0.244	0.250
Indole-3-acetic acid	HP	–0.225	0.258
	HF	–0.065	0.763
Indole-3 propionic acid	HP	0.073	0.717
	HF	0.001	0.741
3-hydroxyindole	HP	0.396	0.041
	HF	0.364	0.081
Hippuric acid	HP	0.064	0.753
	HF	–0.410	0.047

**Table 4:** Unadjusted linear regression models for association between gut-related metabolites with ALSTI/BMI.

Predictor	Group	β (Standardized)	*p*-Value
Hippuric acid	HP	–0.166	0.416
	HF	0.013	0.956
Indole-3-acetic acid	HP	0.132	0.522
	HF	–0.129	0.567
Indole-3 propionic acid	HP	0.001	0.154
	HF	0.001	0.645
3-hydroxyindole	HP	0.066	0.750
	HF	–0.305	0.168
Sarcosine	HP	–0.379	0.056
	HF	–0.068	0.764
Sarcosinamide	HP	–0.012	0.955
	HF	0.087	0.699

**Table 5:** Unadjusted linear regression models for association between mitochondrial metabolites with GDF-15 in HP and HF groups.

Predictor	Group	β (Standardized)	*p*-Value
Pyruvic acid	HP	0.470	0.024
	HF	0.695	<0.001
Fumaric acid	HP	–0.161	0.463
	HF	0.729	<0.001
Citric acid	HP	0.151	0.491
	HF	–0.133	0.566
Glutamic acid	HP	0.338	0.114
	HF	–0.104	0.653
2-Oxoglutaric (glutaric) acid	HP	0.128	0.561
	HF	0.304	0.181
Malic acid	HP	0.097	0.661
	HF	0.296	0.193
Proline	HP	0.287	0.184
	HF	0.305	0.179

**Table 6:** Unadjusted linear regression models for association between mitochondrial metabolites with ALSTI/BMI in HP and HF groups.

Predictor	Group	β (Standardized)	*p*-Value
Pyruvic acid	HP	–0.374	0.060
	HF	–0.184	0.414
Fumaric acid	HP	0.022	0.915
	HF	–0.053	0.816
Citric acid	HP	0.259	0.202
	HF	0.178	0.428
Glutamic acid	HP	–0.101	0.622
	HF	0.018	0.938
Malonic acid	HP	0.239	0.239
	HF	–0.215	0.336

**Table 7:** Unadjusted linear regression models for association between mitochondrial metabolites with total fat mass (kg) in HF and HF groups.

Predictor	Group	β (Standardized)	*p*-Value
Pyruvic acid	HP	0.325	0.098
	HF	0.223	0.294
Fumaric acid	HP	0.143	0.476
	HF	–0.191	0.371
Citric acid	HP	–0.248	0.212
	HF	–0.162	0.449
Glutamic acid	HP	0.091	0.651
	HF	–0.003	0.987
2-Oxoglutaric (glutaric) acid	HP	0.161	0.423
	HF	–0.056	0.794
Malonic acid	HP	–0.018	0.930
	HF	0.064	0.767

## Data Availability

The data presented in this study are available on request from the corresponding author. The data are not publicly available due to privacy or ethical restrictions.

## Supplementary Materials

The following supporting information can be downloaded at: https://www.mdpi.com/article/10.3390/antiox15020199/s1, Table S1: Key characteristics of study participants with heart failure and healthy controls.

## References

[1] Davidson S.M., Andreadou I., Antoniades C., Bartunek J., Basso C., Brundel B.J.J.M., Byrne R.A., Chiva-Blanch G., da Costa Martins P., Evans P.C. (2025). Opportunities and challenges for the use of human samples in translational cardiovascular research: A scientific statement of the ESC Working Group on Cellular Biology of the Heart, the ESC Working Group on Cardiovascular Surgery, the ESC Council on Basic Cardiovascular Science, the ESC Scientists of Tomorrow, the European Association of Percutaneous Cardiovascular Interventions of the ESC, and the Heart Failure Association of the ESC. Cardiovasc. Res..

[2] di Candia A.M., de Avila D.X., Moreira G.R., Villacorta H., Maisel A.S. (2021). Growth differentiation factor-15, a novel systemic biomarker of oxidative stress, inflammation, and cellular aging: Potential role in cardiovascular diseases. Am. Heart J. Plus.

[3] Meng X., Li Y., Meng L., Yang C., Xia C., Wang X., Wang F. (2025). Growth Differentiation Factor 15 Inhibits Cardiac Fibrosis, Oxidative Stress, Inflammation, and Apoptosis in a Rat Model of Heart Failure with Preserved Ejection Fraction. Front. Biosci..

[4] Kozhevnikova M.V., Belenkov Y.N., Shestakova K.M., Ageev A.A., Markin P.A., Kakotkina A.V., Korobkova E.O., Moskaleva N.E., Kuznetsov I.V., Khabarova N.V. (2025). Metabolomic profiling in heart failure as a new tool for diagnosis and phenotyping. Sci. Rep..

[5] Huang R., Shen Z.-Y., Huang D., Zhao S.-H., Dan L.-X., Wu P., Tang Q.-Z., Ma Z.-G. (2025). Microbiota-indole-3-propionic acid-heart axis mediates the protection of leflunomide against αPD1-induced cardiotoxicity in mice. Nat. Commun..

[6] Liu G., Nguyen N.Q.H., Wong K.E., Agarwal S.K., Boerwinkle E., Chang P.P., Claggett B.L., Loehr L.R., Ma J., Matsushita K. (2024). Metabolomic Association and Risk Prediction With Heart Failure in Older Adults. Circ. Heart Fail..

[7] Schiattarella G.G., Kontaridis M.I. (2025). Interorgan Crosstalk in Heart Failure and Cardiometabolic Diseases: A Compendium. Circ. Res..

[8] Tang W.H.W., Li D.Y., Hazen S.L. (2019). Dietary metabolism, the gut microbiome, and heart failure. Nat. Rev. Cardiol..

[9] Lavelle A., Sokol H. (2020). Gut microbiota-derived metabolites as key actors in inflammatory bowel disease. Nat. Rev. Gastroenterol. Hepatol..

[10] Wang Y.-C., Koay Y.C., Pan C., Zhou Z., Tang W., Wilcox J., Li X.S., Zagouras A., Marques F., Allayee H. (2024). Indole-3-Propionic Acid Protects Against Heart Failure With Preserved Ejection Fraction. Circ. Res..

[11] Xing P.Y., Agrawal R., Jayaraman A., Martin K.A., Zhang G.W., Ngu E.L., Faylon L.E., Kjelleberg S., Rice S.A., Wang Y. (2024). Microbial Indoles: Key Regulators of Organ Growth and Metabolic Function. Microorganisms.

[12] Sun H., Sun K., Tian H., Chen X., Su S., Tu Y., Chen S., Wang J., Peng M., Zeng M. (2024). Integrated metagenomic and metabolomic analysis reveals distinctive stage-specific gut-microbiome-derived metabolites in intracranial aneurysms. Gut.

[13] Agus A., Clément K., Sokol H. (2021). Gut microbiota-derived metabolites as central regulators in metabolic disorders. Gut.

[14] Martínez-Reyes I., Chandel N.S. (2020). Mitochondrial TCA cycle metabolites control physiology and disease. Nat. Commun..

[15] Furukawa S., Fujita T., Shimabukuro M., Iwaki M., Yamada Y., Nakajima Y., Nakayama O., Makishima M., Matsuda M., Shimomura I. (2004). Increased oxidative stress in obesity and its impact on metabolic syndrome. J. Clin. Investig..

[16] Trøseid M., Andersen G., Broch K., Hov J.R. (2020). The gut microbiome in coronary artery disease and heart failure: Current knowledge and future directions. EBioMedicine.

[17] Van Hul M., Cani P.D. (2023). The gut microbiota in obesity and weight management: Microbes as friends or foe?. Nat. Rev. Endocrinol..

[18] Takaoka M., Tadross J.A., Al-Hadithi A., Zhao X., Villena-Gutiérrez R., Tromp J., Absar S., Au M., Harrison J., Coll A.P. (2024). GDF15 antagonism limits severe heart failure and prevents cardiac cachexia. Cardiovasc. Res..

[19] Kamper R.S., Nygaard H., Praeger-Jahnsen L., Ekmann A., Ditlev S.B., Schultz M., Hansen S.K., Hansen P., Pressel E., Suetta C. (2024). GDF-15 is associated with sarcopenia and frailty in acutely admitted older medical patients. J. Cachexia Sarcopenia Muscle.

[20] McDonagh T.A., Metra M., Adamo M., Gardner R.S., Baumbach A., Böhm M., Burri H., Butler J., Čelutkienė J., Chioncel O. (2021). 2021 ESC Guidelines for the diagnosis and treatment of acute and chronic heart failure. Eur. Heart J..

[21] Prokopidis K., Farahani S.J., Altinpinar B.G., Khaiyat O., Burke A., Nortcliffe A., Lip G.Y.H., Sankaranarayanan R., Muhamadali H., Isanejad M. (2025). Heart failure with physical frailty is associated with inflammation, insulin resistance, GDF-15 and impaired energy and amino acid metabolism. medRxiv.

[22] Dunn W.B., Wilson I.D., Nicholls A.W., Broadhurst D. (2012). The importance of experimental design and QC samples in large-scale and MS-driven untargeted metabolomic studies of humans. Bioanalysis.

[23] Visser M., Pahor M., Tylavsky F., Kritchevsky S.B., Cauley J.A., Newman A.B., Blunt B.A., Harris T.B. (2003). One- and two-year change in body composition as measured by DXA in a population-based cohort of older men and women. J. Appl. Physiol..

[24] Asnicar F., Manghi P., Fackelmann G., Baldanzi G., Bakker E., Ricci L., Piccinno G., Piperni E., Mladenovic K., Amati F. (2025). Gut micro-organisms associated with health, nutrition and dietary interventions. Nature.

[25] Sugiyama K., Starling N., Chau I. (2025). New Horizons with Growth Differentiation Factor 15 in Oncology: From Cancer Cachexia and Tumour Immunity to Novel Therapeutic Strategies. Curr. Oncol..

[26] Sharma A., Stevens S.R., Lucas J., Fiuzat M., Adams K.F., Whellan D.J., Donahue M.P., Kitzman D.W., Piña I.L., Zannad F. (2017). Utility of Growth Differentiation Factor-15, A Marker of Oxidative Stress and Inflammation, in Chronic Heart Failure: Insights From the HF-ACTION Study. JACC Heart Fail..

[27] Lewis G.A., Rosala-Hallas A., Dodd S., Schelbert E.B., Williams S.G., Cunnington C., McDonagh T., Miller C.A. (2022). Characteristics Associated With Growth Differentiation Factor 15 in Heart Failure With Preserved Ejection Fraction and the Impact of Pirfenidone. J. Am. Heart Assoc..

[28] Prokopidis K., Morwani-Mangnani J., McDowell G., Lip G.Y.H., Venturelli M., Sankaranarayanan R., Isanejad M. (2024). Sarcopenia is linked to higher levels of B-type natriuretic peptide and its N-terminal fragment in heart failure: A systematic review and meta-analysis. Eur. Geriatr. Med..

[29] Liu Y., Ge M., Xiao X., Lu Y., Zhao W., Zheng K., Yu K., He Y., Zhong Q., Zhou L. (2025). Sarcosine decreases in sarcopenia and enhances muscle regeneration and adipose thermogenesis by activating anti-inflammatory macrophages. Nat. Aging.

[30] Cuesta S., Burdisso P., Segev A., Kourrich S., Sperandio V. (2022). Gut colonization by Proteobacteria alters host metabolism and modulates cocaine neurobehavioral responses. Cell Host Microbe.

[31] Zelicha H., Kloting N., Kaplan A., Yaskolka Meir A., Rinott E., Tsaban G., Chassidim Y., Bluher M., Ceglarek U., Isermann B. (2022). The effect of high-polyphenol Mediterranean diet on visceral adiposity: The DIRECT PLUS randomized controlled trial. BMC Med..

[32] Li M., Wang X., Zeng N., Wu Z., Yu C., Sun D., Liu Y., Cao D., Zhang P., Yang L. (2025). Effect of Bariatric Surgery on Non-alcoholic Fatty Liver Disease: An Exploratory Metabolomics and Validation Study. Obes. Surg..

[33] Vacca A., Schiattarella G.G. (2024). From Gut to Heart: Role of Indole-3-Propionic Acid in HFpEF. Circ. Res..

[34] Mirkowski K., Vellone E., Żółkowska B., Jędrzejczyk M., Czapla M., Uchmanowicz I., Uchmanowicz B. (2025). Frailty and Heart Failure: Clinical Insights, Patient Outcomes and Future Directions. Card. Fail. Rev..

[35] Liu H., Wang S., Wang J., Guo X., Song Y., Fu K., Gao Z., Liu D., He W., Yang L.L. (2025). Energy metabolism in health and diseases. Signal Transduct. Target. Ther..

[36] Hinton A., Claypool S.M., Neikirk K., Senoo N., Wanjalla C.N., Kirabo A., Williams C.R. (2024). Mitochondrial Structure and Function in Human Heart Failure. Circ. Res..

[37] Conte M., Sabbatinelli J., Chiariello A., Martucci M., Santoro A., Monti D., Arcaro M., Galimberti D., Scarpini E., Bonfigli A.R. (2021). Disease-specific plasma levels of mitokines FGF21, GDF15, and Humanin in type II diabetes and Alzheimer’s disease in comparison with healthy aging. Geroscience.

[38] Chen J., Kastroll J., Bello F.M., Pangburn M.M., Murali A., Smith P.M., Rychcik K., Loughridge K.E., Vandevender A.M., Dedousis N. (2025). Skeletal muscle mitochondrial dysfunction is associated with increased Gdf15 expression and circulating GDF15 levels in aged mice. Sci. Rep..

[39] Lena A., Anker M.S., Springer J. (2020). Muscle Wasting and Sarcopenia in Heart Failure-The Current State of Science. Int. J. Mol. Sci..

