# Supplementary material for: Associations Between Gut Microbiota and Mitochondrial Metabolites, with Growth Differentiation Factor-15 as a Marker of Oxidative Stress in Heart Failure vs. Healthy Ageing
Source: Antioxidants (Basel). 2026 Feb 2;15(2):199. doi: 10.3390/antiox15020199 (PMC12938193; doi:10.3390/antiox15020199)
Supplement: Supplementary file 1 [file antioxidants-15-00199-s001.zip › antioxidants-4076335-supplementary.pdf]

Supplementary Table S1. Key characteristics of study participants with heart failure and healthy controls

Characteristics<br>Mean ± SD/Median [IQR]/ n (%)	Healthy Control<br>(n = 29)	Heart Failure<br>(n = 25)	p-value*
Demographics			
Age, years	67.9 ± 11.1	68.0 ± 10.0	0.97
BMI, kg/m^2	25.5 [23.6 – 27.3]	28.5 [26.0 –35.6]	0.005*
Female number (%)	15 (51.7)	6 (24.0)	0.04*
NYHA classification	-		
Class I	-	6 (24.0)	
Class II	-	14 (56.0)	
Class III	-	5 (20.0)	
Class IV	-	0 (0)	-
Heart Failure type	-		
HFrEF	-	18 (72.0)	
HFmrEF	-	0 (0.0)	
HFpEF	-	7 (28.0)	-
